# The Use of Self-Reported Functional Limitation to Examine Pregnancy and Reproductive Health Experiences in a National Sample of Women

**DOI:** 10.1089/whr.2021.0015

**Published:** 2022-04-08

**Authors:** Caitlin A. Ward, Katherine D. Goss, John S. Angles, Margaret A. Turk

**Affiliations:** ^1^ College of Medicine, SUNY Upstate Medical University, Syracuse, New York, USA.; ^2^ Department of Physical Medicine and Rehabilitation, SUNY Upstate Medical University, Syracuse, New York, USA.; ^3^ Department of Epidemiology and Biostatistics, University at Albany School of Public Health, State University of New York, Rensselaer, New York, USA.

**Keywords:** reproductive health, pregnancy, infertility, disability, functional limitation

## Abstract

**Context:**

A lack of consensus in the literature examining reproductive health experiences of women with disability prevails, in part, due to various operational definitions of disability.

**Methods:**

Results from the 2015–2016 National Health and Nutrition Examination Survey (NHANES) were utilized to assess reproductive health, disability, and demographic variables among women aged 20–44. Disability was assessed using the six functional limitation subgroups. Analyses included modified Poisson regression and negative binomial regression.

**Results:**

One hundred eighty-two (14%) women reported having any functional limitation. Women with at least one functional limitation (WWFL) were significantly more likely than women without a functional limitation (WWOFL) to have had a hysterectomy and had more cesarean deliveries. WWFL did not differ significantly from WWOFL in key pregnancy outcomes (ever been pregnant, number of pregnancies, or number of unsuccessful pregnancies). A high degree of overlap between mobility and self-care (66.1%), cognitive and independent living (61%), and mobility and independent living (37.4%) limitations was found.

**Conclusions:**

This work summarizes key reproductive health variables among US women of reproductive age and contextualizes disability experiences through subgroup and overlap analysis. Subgroup analysis results demonstrate the need for detailed operational definitions of disability to accurately capture experiences of women with different limitations, and overlap analysis indicates the interconnectedness of limitations among this group. Findings call for future exploration of reproductive health-related similarities and differences between WWD and women without disability, and employment of detailed operational definitions of disability.

## Introduction

In 1990, the Americans with Disabilities Act (ADA) was signed into law. This historical policy makes it illegal for those with power in society—employers, landlords, doctors, and others—to discriminate against people with disability. Among other protections, the ADA requires all doctors' offices provide meaningful access to their services for people with disability, regardless of whether the disability requires accommodations.^1^ The implementation of this policy, along with the growing momentum of the disability and reproductive rights movements, has led to increased reproductive autonomy for women with disability (WWD).

Currently, there is a lack of consensus in the literature surrounding pregnancy and reproductive health outcomes among WWD. Several studies cite a decreased rate of current pregnancy as well as an increased risk of complications in pregnancy and birth outcomes among WWD,^2–5^ while others support a similar rate of pregnancy among WWD, and no difference in current pregnancy, live births, or abortions.^6,7^ Factors influencing this lack of consensus in the literature have not been sufficiently explored.

The heterogeneity of experiences among women with different types of disability, combined with the broad range of disability definitions utilized in existing work, are two factors that may contribute to this lack of consensus. For example, some articles reflect experiences of a very specific subgroup of people with disability (*e.g.*, spina bifida, cognitive disability) and thus, are not generalizable to all individuals with disability. In turn, however, broader definitions of disability—like the six functional disability questions developed by the Washington Group,^8^ utilized by a growing number of national level surveys—are also used differently across relevant literature.

Importantly, women may manifest more than one type of functional limitation (80% of women who reported a self-care limitation also reported a mobility limitation), signaling that utilized disability definitions and subgroups may not be mutually exclusive.^9^

In addition to literature examining pregnancy experiences of WWD, a recent study has extended potential reproductive health differences among WWD to the field of fecundity and infertility. This study, utilizing the six functional limitation questions, found that women with cognitive limitation had significantly decreased fecundity compared to women without a limitation.^10^ No significant difference in fecundity was found among women with a sensory or physical disability. This study confirms and extends support for a decreased rate of pregnancy for women with this type of disability. Despite support for a decreased fecundity and some support for an increased rate of complications in pregnancy, WWD experience disparities in the receipt of sexual and reproductive health care.^11^ With growing evidence that WWD want to become parents, and are engaging in sexual activity at rates similar to women of reproductive age without disability,^12^ reproductive and sexual health education for this population may be of increasing importance.

This study explores pregnancy and reproductive health experiences among a national sample of WWD. The utilized data source, the National Health and Nutrition Examination Survey (NHANES), provides information on both topics of interest. Through subgroup and overlap analysis, this study also seeks to contextualize the presence of disability among this specific population. Subgroup analysis promotes an enhanced understanding of the experiences of women in specific functional limitation categories, and how the findings for these subgroups compare to the findings for all women with at least one functional limitation (WWFL), while overlap analysis enhances understanding of the interconnectedness of functional limitations among this group.

Thus, this report has several primary research objectives: (1) examine differences in reproductive health and pregnancy outcomes (*i.e.*, having ever been pregnant, number of pregnancies, and help seeking) across women with or without a functional limitation, 2) examine differences in reproductive health and pregnancy outcomes among subgroups of women with a functional limitation, and 3) describe presence and interconnectedness of disability among reproductive aged women in the United States.

## Methods

Data from the 2015–2016 NHANES were utilized for analyses. NHANES is conducted annually by the Centers for Disease Control and Prevention (CDC), and uses a complex samples analysis to collect health data that is representative of the entire noninstitutionalized population of the United States.^13^ Data from the demographics, reproductive health, and disability modules were used in this study. Responses from female participants between the ages of 20 and 44 were used for analysis, as the reproductive health module from NHANES reflects responses from women in this age range.^14^

As this study used previously collected and de-identified public data, Institutional Review Board approval from Upstate Medical University was waived, as it was determined that the project did not meet the definition of human subjects research.

### Data items

For all variables, responses that were recorded as “missing,” “don't know,” or “refused” were excluded from analyses.

#### Demographics

Data on race, age, education level, annual household income, and marital status were reported.

#### Disability

The six Washington Group questions representing functional limitation are used by NHANES to indicate individuals with disability.^8^

These questions ask the respondent whether they have one of six functional limitations: hearing (“Are you deaf or do you have serious difficulty hearing?”), vision (“Are you blind or do you have serious difficulty seeing even when wearing glasses?”), cognitive (“Because of a physical, mental, or emotional condition, do you have serious difficulty concentrating, remembering, or making decisions?”), mobility (“Do you have serious difficulty walking or climbing stairs?”), self-care (“Do you have difficulty dressing or bathing?”), or independent living (“Because of a physical, mental, or emotional condition, do you have difficulty doing errands alone?”).

Each question was asked and reported independently, with respondents reporting either having or not having the given limitation. Thus, a woman could be included in more than one functional limitation category if she responded “yes” to more than one of the above questions. NHANES does not ask survey participants about the time of onset of their functional limitation. Therefore, it is not possible to discern the timeline of onset of functional limitation and occurrence of the various reproductive health outcomes, including pregnancy.

Two other additional variables were calculated using this data. A variable indicating a sensory limitation was created for respondents who stated they had difficulty with hearing and/or seeing. This categorization is consistent with previous research, which used the six functional limitation questions to compare reproductive health outcomes in women with and without disability.^10^ This variable, in combination with the other functional limitation categories, was used for subgroup and overall analysis.

A summary variable was also created to differentiate women who self-reported having any one of the above listed functional limitations (WWFL) from women who had none of the above listed functional limitations (WWOFL). This grouping is also in line with past interpretation of functional limitation status to define disability.^12,15^ The data for each disability type (sensory, cognitive, mobility, self-care, and independent living) were analyzed individually *via* a subgroup analysis.

In addition, the percentage of overlap between certain functional limitations (mobility-independent living, mobility-self-care, and cognitive-independent living) was calculated, along with the percent of women who indicated having only one type of functional limitation. A respondent was included in the overlap group if she reported having both limitations—for example, mobility and independent living; mobility and self-care; and/or cognitive and independent living. Pairings are not exhaustive, but rather look to assess limitation groups that commonly coincide.

The purpose of this overlap analysis was to contextualize and describe disability among this population, rather than to combine and analyze reproductive health outcomes for women with multiple limitations. Regardless, relatively low levels of overlap in each overlap grouping discouraged statistical merging of functional limitation categories. The highest percentage of overlap, 66.1% was notably lower than overlap percentages used to merge limitation categories in a past study.^9^

#### Reproductive health

Primary outcome variables included having ever been pregnant, number of past pregnancies, number of live births, having tried for a year to become pregnant, and seeking medical help with fertility. In addition, a variable indicating the number of past unsuccessful pregnancies was calculated by subtracting the number of live births from the total number of pregnancies for each respondent. This variable is operationally defined as the number of pregnancies that did not result in live birth.

Other reproductive health variables included having had a hysterectomy, having both ovaries removed, and the participant being told they had diabetes during their pregnancy. A new binary (yes/no) variable for diabetes was created, wherein women who responded “yes” or “borderline” to the question of whether they were told they had diabetes during pregnancy were recorded as “yes” responses.

### Statistical analyses

All analyses were conducted with IBM SPSS Version 24, SAS Version 9.4, or STATA/BE Version 17. To account for the complex sampling methods utilized by NHANES, all calculations were made using the IBM Complex Samples within, following IBM manual recommendations,^16^ or survey estimation commands in STATA, following manual recommendations from IBM and STATA, respectively. Sample weights provided in the NHANES 2015–2016 dataset were used in complex sample calculations. Thus, estimated values presented in this report are generalizable to all noninstitutionalized reproductive aged women (ages 20–44) living in the United States.

For all variables, responses that were recorded as “missing,” “don't know,” or “refused” were excluded from analyses. Descriptive values were calculated for all variables. Rate ratios and adjusted relative risks are reported to describe relationships between variables. Chi square analyses and *t*-tests were conducted to assess if there were significant differences in demographic data between WWFL and women without a functional limitation (WWOFL). Relative risk was assessed *via* a modified Poisson regression where appropriate. Negative binomial regression was used to assess count outcomes. Five demographic variables (race, education, marital status, annual household income, and age) associated with reproductive outcomes were included in all final models.

## Results

A total of 1,288 responses were collected from women between the ages of 20 and 44. Forty-eight of these responses did not have a health examination completed by the NHANES Medical Examination Center (MEC), and thus were unable to be weighed and included in analysis. Thus, a total of 1,240 positively weighted responses were received and included in analyses. These responses are representative of all noninstitutionalized women in this age range in the United States.

Significant differences in education level, marital status, and annual household income existed between women with and without any reported functional limitation (WWFL and WWOFL, respectively). WWFL were less likely to have graduated college; were less likely to be married; and less likely to have an annual household income above $65,000 (Table 1).

**Table 1.** Demographics

**Variable**	**Observed frequency**	**Percent (SE)** ^a^	**Percent WWFL (SE)** ^a^	**Percent WWOFL (SE)** ^a^
Race				
Non-Hispanic white	241	54.3 (4.60)	51.3 (6.77)	54.7 (4.54)
Non-Hispanic black	282	13.8 (2.98)	16.1 (3.78)	13.4 (2.96)
Mexican American	242	12.2 (2.87)	13.66 (4.07)	11.9 (2.78)
Non-Hispanic Asian	170	7.30 (1.36)	5.03 (1.27)	7.67 (1.47)
Other Hispanic	152	8.01 (1.45)	7.88 (1.98)	8.03 (1.45)
Other/Multiracial	53	4.46 (0.53)	6.06 (1.90)	4.20 (0.66)
Education level				
<9th grade	97	5.21 (1.14)	8.04 (2.75)	4.74 (1.07)
9–11th grade (incl. 12th grade no diploma)^*^	106	6.50 (1.07)	12.6 (1.93)	5.50 (1.07)
HS graduate/GED^*^	227	16.5 (1.38)	25.2 (3.49)	15.1 (1.68)
Some college/AA degree	441	36.5 (2.28)	34.4 (4.83)	36.9 (2.57)
College graduate or above^*^	369	35.3 (2.89)	19.8 (3.91)	37.8 (2.91)
Marital status				
Married^*^	540	45.2 (1.98)	27.7 (4.78)	48.1 (2.03)
Never married^*^	380	29.2 (2.26)	38.9 (5.57)	27.6 (2.19)
Living with partner	190	15.3 (1.44)	13.7 (2.51)	15.5 (1.54)
Divorced	83	6.86 (1.01)	11.1 (2.02)	6.16 (1.22)
Separated^*^	41	2.66 (0.62)	6.62 (2.53)	2.02 (0.46)
Widowed	6	0.81 (0.42)	1.87 (1.87)	0.64 (0.41)
Annual household income				
<$20,000^*^	198	12.6 (1.42)	26.6 (3.93)	10.3 (1.33)
$20,000–$64,999	559	44.0 (2.40)	45.6 (5.06)	43.7 (2.94)
>$65,000^*^	418	43.4 (2.67)	27.8 (3.51)	46.0 (2.84)

**Variable**		**Mean estimate, total (SE)**	**WWFL, mean (SE)**	**WWOFL, mean (SE)**
Age (years)				
		31.89 (0.334)	31.81 (0.804)	31.90 (0.309)

^a^ Value is estimated to reflect the study population (*i.e.*, women aged 20–44 years living in the United States).

^*^ Indicates statistically significant difference (*p* < 0.05) between WWFL and WWOFL.

SE, standard error; WWFL, women with functional limitation; WWOFL, women without functional limitation.

### Functional limitation status

A total of 182 respondents (14%) reported a serious difficulty in at least one of the six categories, indicating at least one functional limitation. The most commonly reported functional limitation was cognitive (7.4%). Mobility limitation was the second most commonly reported (4.4%), followed by sensory (4.3%), independent living (4.1%), and self-care (0.9%). See Table 2 for a complete breakdown of functional limitation outcomes.

**Table 2.** Functional Limitation and Reproductive Health Variables

**Limitation type**	**Observed frequency**	**Percent (SE)** ^a^	
Cognitive	95	7.44 (0.85)	
Mobility	63	4.38 (0.73)	
Sensory	55	4.28 (0.66)	
Independent living	53	4.12 (0.68)	
Self-care	16	0.88 (0.22)	
*Reported any functional limitation*	*182*	*14.0 (1.21)*	

	**Observed frequency**	**Percent (SE)** ^a^	**Mean (SE)** ^a^
Pregnancy and reproductive health			
Ever been pregnant?	762	68.1 (3.02)	—
Tried for a year to become pregnant?	116	11.5 (1.10)	—
Visited a doctor b/c unable to become pregnant?	71	8.27 (1.10)	—
Had a hysterectomy?	37	4.07 (0.76)	—
Had both ovaries removed?	10	1.39 (0.50)	
How many times have you been pregnant?	—	—	2.97 (0.09)
Number of unsuccessful pregnancies	—	—	0.85 (0.06)
Deliveries			
How many deliveries resulted in a live birth?			2.26 (0.05)

^a^ Value is estimated to reflect the study population (*i.e.*, women aged 20–44 years living in the United States).

**FIG. 1.**
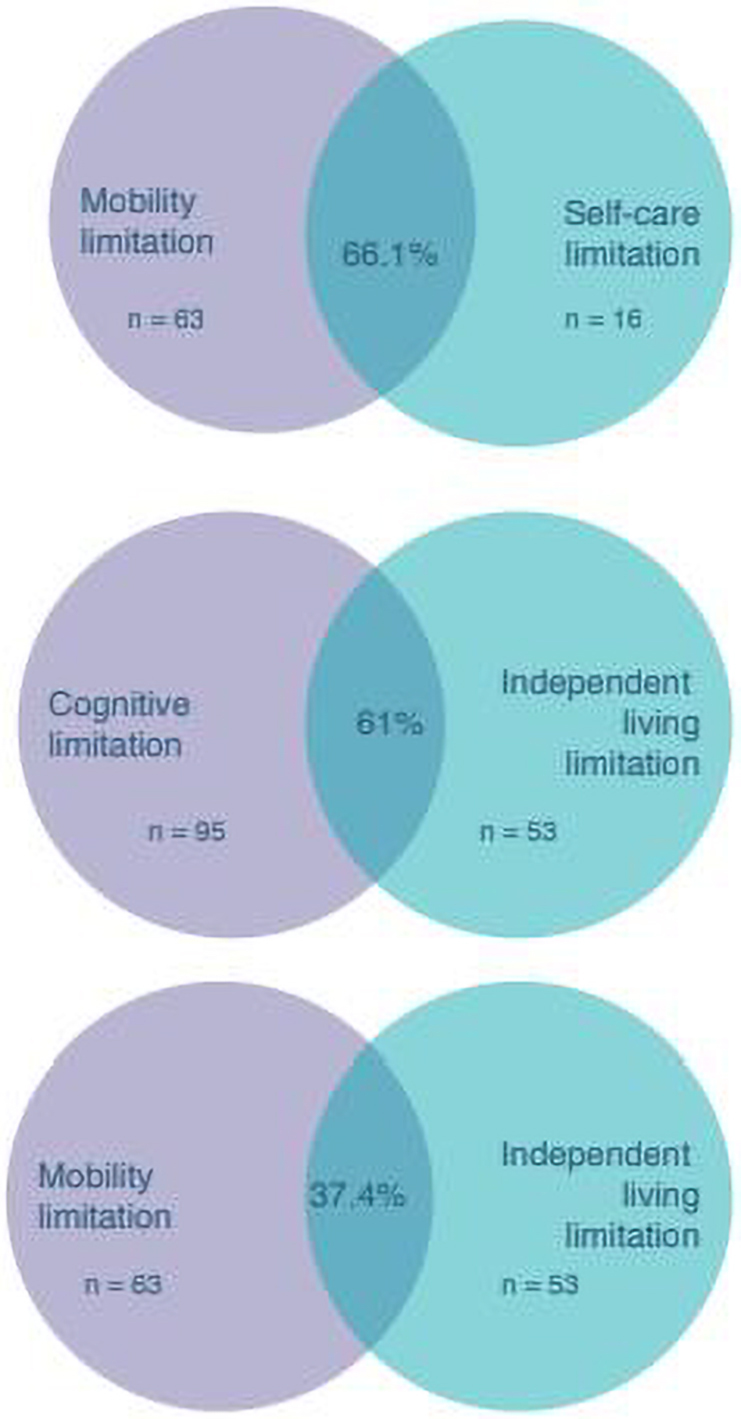
Venn diagram representation of percent overlap between women with different functional limitations.

The overlap analysis found that among women who reported a mobility or self-care limitation, 66.1% of women reported having both limitations, and 37.4% of women reported both a mobility and independent living limitation. Sixty-one percent of women reported both a cognitive and independent living limitation (Fig. 1). When analyzed independently, cognitive limitation was the most commonly reported (3.8%), followed by mobility (1.7%), hearing (1.3%), independent living (1.3%), vision (1.2%), and self-care (0.1%). There were no respondents who reported both a hearing and vision limitation, with no other limitations.

### Reproductive health and pregnancy outcomes

An estimated 68% of the study population had ever been pregnant. On average, respondents had 2.97 (standard error [SE] = 0.09) pregnancies and 0.85 (SE = 0.06) pregnancies that were unsuccessful. An estimated 11.5% of the study population had tried for a year to become pregnant, and 8.3% of the study population had visited a doctor because they were unable to become pregnant (Table 2).

### Comparison of reproductive health and pregnancy outcomes between groups

In terms of key pregnancy outcomes, WWFL were not significantly different from WWOFL. WWFL did not have a significantly different risk of having ever been pregnant compared to WWOFL (relative risk [RR]) = 1.08, 95% confidence interval [CI]: 0.98–1.19). Further, WWFL were not significantly different from WWOFL in number of past pregnancies (RR = 0.98, 95% CI: 0.86–1.11), or in number of unsuccessful pregnancies (RR = 0.99, 95% CI: 0.7–1.39). WWFL did not have a significantly higher or lower risk of being told that they had diabetes during pregnancy, compared to WWOFL (RR = 1.21, 95% CI: 0.74–1.97).

WWFL were significantly more likely to have had a hysterectomy (RR = 2.42, 95% CI: 1.02–5.7). WWFL also had a significantly higher risk of having cesarean procedures than WWOFL (RR = 1.45, 95% CI: 1.13–1.85; Table 3).

**Table 3.** Final Adjusted^a^ Pregnancy and Reproductive Health Experiences for Women with at Least One Functional Limitation

**Variable** ^a^	**Relative risk**	**Lower 95% CI**	**Upper 95% CI**	**% of WWFL** ^b^	**% of WWOFL** ^b^
Ever been pregnant	1.08	0.98	1.19	77.2	66.6
Tried for a year to become pregnant	1.33	0.7	2.53	12.9	11.3
Visited a doctor because unable to become pregnant	1.08	0.43	2.69	6.8	8.5
Told they had diabetes during pregnancy	1.21	0.74	1.97	13.4	11.9
Hysterectomy	2.42^*^	1.02	5.7	9.1	3.2
Ovaries removed	3.6	0.52	24.8	3.6	1.0

**Variable** ^a^	**Rate ratio**	**Lower CI**	**Upper CI**	**Mean (SE) of WWFL** ^b^	**Mean (SE) of WWOFL** ^b^
No. of past pregnancies	0.98	0.86	1.11	3.12 (0.21)	2.94 (0.08)
No. of unsuccessful pregnancies	0.99	0.7	1.39	0.89 (0.15)	0.85 (0.05)
No. of vaginal deliveries	0.86	0.68	1.1	1.57 (0.18)	1.61 (0.06)
No. of C-sections	1.45^*^	1.13	1.85	1.15 (0.12)	0.78 (0.08)

^a^ All variables adjusted for race, education, marital status, annual household income, and age.

^b^ Value is estimated to reflect the study population (*i.e.*, women aged 20–44 years living in the United States).

^*^ Statistically significant result at *p* < 0.05.

CI, confidence interval.

### Functional limitation subgroup analysis

After adjusting for all confounders, no significant differences existed for major reproductive health outcomes (*i.e.*, having ever been pregnant, number of past pregnancies, and number of unsuccessful pregnancies) between women who did or did not report a specific limitation (Table 4). Women who reported a mobility limitation had a significantly higher risk of being told they had diabetes during pregnancy, compared to women without this limitation (RR = 2.13, 95% CI: 1.14–3.97).

**Table 4.** Final Adjusted^a^ Pregnancy and Reproductive Health Outcomes by Functional Limitation Subgroup

**Variable** ^a^	**Limitation**	**Rate ratio**	**Lower 95% CI**	**Upper 95% CI**	**Mean (SE) of WWFL** ^b^	**Mean (SE) of WWOFL** ^b^
No. of past pregnancies	…cognitive	0.89	0.76	1.03	3.02 (0.22)	2.97 (0.084)
	… mobility	1.11	0.97	1.26	3.6 (0.26)	2.93 (0.085)
	…independent living	0.97	0.74	1.27	3.35 (0.49)	2.95 (0.084)
	…sensory	0.96	0.82	1.14	3.19 (0.33)	2.96 (0.081)
	… self-care	1.23	0.98	1.54	4.14 (0.65)	2.96 (0.084)
No. of unsuccessful pregnancies	… cognitive	0.82	0.53	1.27	0.80 (0.19)	0.86 (0.05)
	… mobility	1.14	0.67	1.94	1.01 (0.24)	0.84 (0.057)
	… independent living	0.96	0.53	1.73	0.89 (0.27)	0.85 (0.054)
	…sensory	1.02	0.63	1.65	0.92 (0.22)	0.85 (0.056)
	… self-care	1.04	0.44	2.48	0.99 (0.44)	0.85 (0.055)

**Variable**	**Limitation**	**Relative risk**	**Lower CI**	**Upper CI**	**% of WWFL (SE)** ^b^	**% of WWOFL (SE)** ^b^
Ever been pregnant	… cognitive	1.02	0.89	1.15	76.7 (6.64)	67.4 (3.05)
	… mobility	1.06	0.89	1.26	82.1 (6.63)	67.4 (2.97)
	… independent living	0.99	0.84	1.16	75.5 (7.35)	67.8 (3.01)
	…sensory	1.07	0.93	1.25	89.2 (5.19)	67.2 (2.96)
	… self-care	1.15	0.89	1.49	94.6 (0.50)	67.9 (3.08)
Tried for a year to become pregnant	… cognitive	0.74	0.35	1.58	8.24 (2.34)	11.8 (1.25)
	… mobility	1.66	0.62	4.46	17.1 (7.51)	11.2 (1.09)
	… independent living	0.18	0.03	1.31	1.99 (0.38)	11.9 (1.13)
	…sensory	1.72	0.77	3.85	20.1 (8.15)	11.1 (1.02)
	… self-care	2.81	0.62	12.78	26.1 (15.7)	11.4 (1.10)
Visited a doctor because unable to become pregnant	… cognitive	0.56	0.14	2.17	3.35 (1.73)	8.67 (1.29)
	… mobility	1.53	0.37	6.32	11.3 (6.63)	8.11 (1.87)
	… independent living	0.33	0.04	2.83	1.99 (0.38)	8.55 (1.19)
	…sensory	1.73	0.6	5.0	13.5 (6.69)	8.04 (1.09)
	… self-care	4.08	0.48	34.4	20.7 (15.6)	8.15 (1.16)
Diabetes during pregnancy	… cognitive	0.68	0.27	1.72	8.54 (3.52)	12.5 (1.55)
	… mobility	2.13^*^	1.14	3.97	23.8 (5.98)	11.4 (1.33)
	… independent living	1.34	0.45	4.05	16.5 (4.63)	11.9 (1.40)
	…sensory	1.4	0.57	3.44	16.8 (5.30)	11.9 (1.55)
	… self-care	0.89	0.11	7.39	9.38 (8.49)	12.2 (1.30)

^a^ All variables adjusted for race, education, marital status, annual household income, and age.

^b^ Value is estimated to reflect the study population (*i.e.*, women aged 20–44 years living in the United States).

^*^ Statistically significant at *p* < 0.05.

## Discussion

Among reproductive aged women living in the United States captured by the 2015–2016 NHANES, WWFL were not significantly different from WWOFL in key pregnancy outcome variables analyzed by this study (*i.e.*, having ever been pregnant, number of past pregnancies, and number of unsuccessful pregnancies). However, WWFL had a significantly higher risk of having a hysterectomy and had a significantly higher risk of having cesarean procedures than WWOFL.

Subgroup analysis found no significant differences in major reproductive health outcomes between women who did and did not report a specific functional limitation, however, did find that women who reported a mobility limitation were more likely to have been told they had diabetes during pregnancy. Overlap analysis further complicates these findings, as functional limitation subgroups are far from mutually exclusive and real-life experiences of disability and reproductive health outcomes may vary.

### Gynecological procedures

The findings in this article that WWFL had significantly higher risk of having a hysterectomy, and had a significantly higher risk of cesarean procedures than WWOFL, are consistent with prior work that has investigated the topic of disability, reproductive health, and pregnancy in women.^5,17^ A woman may have a hysterectomy because of a medical condition, or as a form of permanent contraception^18^; however, NHANES does not ask survey respondents to disclose why they had a hysterectomy.

Previously, researchers found that women with a disability had higher odds of reporting underlying medical conditions, raising the possibility that WWFL may have higher odds of having a hysterectomy because of health issues.^2^ However, other researchers have found that women with a disability were more likely to undergo a sterilization procedure, including hysterectomy, regardless of health status.^19^

It is also important to note that NHANES is a cross-sectional survey, therefore, it is possible that survey respondents had a hysterectomy before the onset of a functional limitation. Physicians should ensure that WWFL who are considering a hysterectomy as a form of birth control understand all of their contraception options, including long-acting reversible contraceptives, which are highly effective at preventing pregnancy and have fewer complications than sterilization procedures.^20^ Future research should investigate why WWD report more cesarean procedures and hysterectomies, compared to women without disability (WWOD).

### Subgroup analysis

No significant differences in major reproductive health outcomes were found among women who reported different subtypes of functional limitation. Prior research has shown that the odds of experiencing an unintended pregnancy differ by disability type,^9^ and that women with physical, intellectual, and/or sensory disability have different prenatal care experiences.^21^ The present study found that women with a mobility disability had a significantly higher risk of having been told that they have diabetes during pregnancy. WWD do not have a monolithic pregnancy experience, and physicians and other health care providers should anticipate as much diversity in this population as they would among WWOD.

### Overlap analysis

Real-life influence of disability on reproductive health experiences may be further complicated by the intersection of different functional limitation categories. Overlap, or intersection, of different limitations was assessed through report of the six functional limitation questions. Specifically, overlap between mobility and self-care (66.1%), mobility and independent living (37.4%), and cognitive and independent living limitations (61%) were calculated. Cognitive and mobility limitations were most commonly reported whether reported independently (3.8% and 1.7%, respectively) or in conjunction with another limitation. Small percentages of women reported having only a hearing (1.3%), independent living (1.3%), vision (1.2%), or self-care (0.1%) limitation.

Given this, compounding effects of these limitations on real-life experiences of WWD should not be underestimated. While not a focus of this analysis, reproductive health care providers should anticipate how multiple limitations may present in different women, and the potential additive effect of these limitations on reproductive health experiences. The intersectionality between limitations, and the rarity of reporting only one limitation, reiterates that WWD should be viewed holistically by all medical professionals, including obstetrician-gynecologists and other reproductive health care providers.

### The use of functional limitation categories to assess disability

Definitions of disability used in the body of literature assessing reproductive health experiences of individuals in this population vary, which may contribute to a lack of consistency in the literature. For example, one of the main findings of this study—that WWFL did not significantly differ from WWOFL in number of live births—is consistent with the findings in a study that also used functional limitations to operationalize disability,^6^ but are inconsistent with another study which used ICD-9 codes to define intellectual or developmental disability.^3^ Diagnosis codes should acknowledge a broad expectation of levels of function within individuals of each ICD code, and thus the differences in results are not surprising.

This study utilized a subgroup analysis to understand within group differences of WWFL, in an effort to explore the influence of operational definitions of disability. This subgroup analysis provided new insight into important clinical experiences that would be missed through use of a broad functional limitation definition alone. Overlap analysis further suggested that subgroups were far from mutually exclusive, further complicating the selection of appropriate disability definitions.

Future work should consider utilization of detailed operational definitions of disability and analysis of disability or functional limitation subgroups to understand differences across limitation categories. This inclusion may improve the ability of work to accurately examine experiences of individual groups and, thus, the ability of this work to have clinical importance and relevance. With the prominent usage of functional limitation categories to define disability in many national surveys (*e.g.*, National Health Interview Survey, National Survey of Family Growth, and American Community Survey), these observations will become increasingly relevant.

### Strengths and limitations

This study broadens and contextualizes the current body of knowledge regarding disability and reproductive health experiences of reproductive aged women. Use of NHANES strengthens the external validity of findings, as results can be generalized to all women aged 20–44 living in the United States. The use of functional limitation categories to operationally define disability in this study allowed for the analysis of the entirety of the population of women with a functional limitation, as well as experiences of women within specific functional limitation categories.

In conjunction with the subgroup analysis, overlap analysis sought to describe and contextualize disability among this group, adding to the body of knowledge related to disability among an important population. In addition, the inclusion of fecundity and infertility variables allows for an increased understanding of the clinical importance of reproductive and pregnancy experiences of this group.

While a novel contribution to the field, this study is not without limitations. As discussed in the methods section, the use of NHANES cross-sectional data prohibits researchers from establishing a temporal or causative association between variables and limits analysis to already collected data points. Thus, it is possible that reproductive health experiences occurred before the onset of a functional limitation. In addition, utilization of self-reported data introduces bias, and may not fully reflect lived experiences of this population.

Finally, the age limitation imposed by available NHANES data in the reproductive health questionnaire excludes women younger than 20 and older than 44 from these findings. In 2016, the birth rate for women aged 18 and 19 in the United States was 37.5 per 1,000, and for women older than 44 years, it was 0.9 per 1,000 births.^22^ The exclusion of these age groups from analysis may have limited the generalizability of these results. With these limitations in mind, future work would benefit from cohort methodology and the inclusion of detailed covariates, including sexual activity and contraceptive use, and other health variables to produce a complete and comprehensive picture of the lived experiences of this population.

## Conclusions

This study expands current knowledge on the reproductive health experiences of reproductive aged women and contextualizes the presence and influence of disability among this group. Utilization of a national sample of women allows for generalizable conclusions representing reproductive aged women between 20 and 44 years across the United States. No significant differences in pregnancy outcomes (*e.g.*, having ever been pregnant and number of past pregnancies) between women with and without any functional limitation were detected by this study. While this does not mean that real-world differences may not exist, this study suggests similar experiences among WWD and WWOD in some reproductive health outcomes.

Thus, future research should examine similarities between WWD and WWOD, in addition to important and clinically relevant reproductive health differences. Through this exploration, future work can seek to provide the information necessary to educate health providers on how best to address reproductive concerns among WWD and combat present differences and disparities in the receipt of sexual and reproductive health care. Future work must be mindful of operational definitions of disability and implications of utilized methodology.

## Abbreviations Used

ADA: Americans with Disabilities Act
CDC: Centers for Disease Control and Prevention
CI: confidence interval
LARCs: long-acting reversible contraceptives
MEC: Medical Examination Center
NHANES: National Health and Nutrition Examination Survey
RR: relative risk
SE: standard error
WWD: women with disability
WWFL: women with at least one functional limitation
WWOD: women without disability
WWOFL: women without a functional limitation
